# Automating the data extraction process for systematic reviews using GPT-4o and o3

**DOI:** 10.1017/rsm.2025.10030

**Published:** 2025-09-17

**Authors:** Yuki Kataoka, Tomohiro Takayama, Keisuke Yoshimura, Ryuhei So, Yasushi Tsujimoto, Yosuke Yamagishi, Shiro Takagi, Yuki Furukawa, Masatsugu Sakata, Đorđe Bašić, Andrea Cipriani, Pim Cuijpers, Eirini Karyotaki, Mathias Harrer, Stefan Leucht, Ava Homiar, Edoardo G. Ostinelli, Clara Miguel, Alessandro Rodolico, Toshi A. Furukawa

**Affiliations:** 1 Department of Internal Medicine, Kyoto Min-iren Asukai Hospital, Kyoto, Japan; 2 Scientific Research WorkS Peer Support Group (SRWS-PSG), Osaka, Japan; 3 Department of Healthcare Epidemiology, Kyoto University Graduate School of Medicine/School of Public Health, Kyoto, Japan; 4 Department of International and Community Oral Health, Tohoku University Graduate School of Dentistry, Sendai, Japan; 5 Faculty of Medicine, Kyoto University, Kyoto, Japan; 6 Fitting Cloud Inc., Kyoto, Japan; 7 Department of Psychiatry, Okayama Psychiatric Medical Center, Okayama, Japan; 8 CureApp, Inc., Tokyo, Japan; 9 Oku Medical Clinic, Osaka, Japan; 10 Department of Health Promotion and Human Behavior, Kyoto University Graduate School of Medicine/School of Public Health, Kyoto University, Kyoto, Japan; 11 Division of Radiology and Biomedical Engineering, Graduate School of Medicine, The University of Tokyo, Tokyo, Japan; 12 Independent Researcher; 13 Department of Neuropsychiatry, University of Tokyo, Tokyo, Japan; 14 Department of Neurodevelopmental Disorders, Nagoya City University Graduate School of Medical Sciences, Nagoya, Japan; 15 Faculty of Behavioural and Movement Sciences, Clinical Psychology, Vrije Universiteit Amsterdam, Amsterdam, The Netherlands; 16 Department of Psychiatry, University of Oxford, Oxford, UK; 17 Oxford Precision Psychiatry Lab, NIHR Oxford Health Biomedical Research Centre, Oxford, UK; 18 Oxford Health National Health Service Foundation Trust, Warneford Hospital, Oxford, UK; 19 Department of Clinical, Neuro- and Developmental Psychology, WHO Collaborating Center for Research and Dissemination of Psychological Interventions, Amsterdam Public Health Institute, Vrije Universiteit Amsterdam, Amsterdam, The Netherlands; 20 Section for Evidence-Based Medicine in Psychiatry and Psychotherapy, Department of Psychiatry and Psychotherapy, School of Medicine and Health, Technical University of Munich, Munich, Germany; 21 Kyoto University Office of Institutional Advancement and Communications, Kyoto, Japan

**Keywords:** data extraction automation, GPT-4o, large language models, o3, systematic reviews

## Abstract

Large language models have shown promise for automating data extraction (DE) in systematic reviews (SRs), but most existing approaches require manual interaction. We developed an open-source system using GPT-4o to automatically extract data with no human intervention during the extraction process. We developed the system on a dataset of 290 randomized controlled trials (RCTs) from a published SR about cognitive behavioral therapy for insomnia. We evaluated the system on two other datasets: 5 RCTs from an updated search for the same review and 10 RCTs used in a separate published study that had also evaluated automated DE. We developed the best approach across all variables in the development dataset using GPT-4o. The performance in the updated-search dataset using o3 was 74.9% sensitivity, 76.7% specificity, 75.7 precision, 93.5% variable detection comprehensiveness, and 75.3% accuracy. In both datasets, accuracy was higher for string variables (e.g., country, study design, drug names, and outcome definitions) compared with numeric variables. In the third external validation dataset, GPT-4o showed a lower performance with a mean accuracy of 84.4% compared with the previous study. However, by adjusting our DE method, while maintaining the same prompting technique, we achieved a mean accuracy of 96.3%, which was comparable to the previous manual extraction study. Our system shows potential for assisting the DE of string variables alongside a human reviewer. However, it cannot yet replace humans for numeric DE. Further evaluation across diverse review contexts is needed to establish broader applicability.

## Highlights

### What is already known?

- Large language models have shown promise for automating data extraction (DE) in systematic reviews (SRs), but existing approaches often require manual interaction, lack open-source accessibility, and are not extensively tested on independent large-scale datasets.

### What is new?

- An open-source systems using GPT-4o and o3 were developed for automated DE in SRs. The system using GPT-4o achieved 74.4% sensitivity, 68.8% specificity, 69.1% precision, 97.1% variable detection comprehensiveness, and 72.6% accuracy across all variables in the development dataset.
- In a temporal validation dataset, the system using o3 achieved 74.9% sensitivity, 76.7% specificity, 75.7% precision, 93.5% variable detection comprehensiveness, and 75.3% accuracy.
- In an external validation dataset, the system using GPT-4o achieved 96.3%, which was comparable to the previous manual extraction study.

### Potential impact for RSM readers

- The system showed potential for assisting in the extraction of string data in combination with human input, but the performance for numeric DE was still inadequate due to limited accuracy.

## 1 Introduction

Systematic reviews (SRs) play a critical role in evidence-based medicine. They provide comprehensive summaries of existing research on specific clinical questions, which are essential for advancing science. However, they rely on time-consuming systematic processes, often leading to outdated results, thus requiring efficient process improvement.^1^^,^ ^2^

Hence, the SR project requires improvements in workflow efficiencies. While satisfactory results have been reported for the use of machine learning (ML) in updating SR searches,^3^^,^ ^4^ data extraction (DE) tasks remain challenging, even with traditional ML approaches.^5^ Since the advent of ChatGPT in 2022, expectations that large language models (LLMs) will lead to advances in this field have been growing.^6^^–^ ^8^

To date, reported attempts to automate DE using LLMs have several limitations in terms of their reliable implementation in SRs. First, some models do not target specific SR questions.^9^^–^ ^12^ Instead, they extract data on what the original authors regarded as the study-specific “primary outcome,” rather than relying on a specific review question, as typically done in SRs. Other models limit their focus to specific fields like oncology, where DE can sometimes be relatively straightforward due to lower heterogeneity in core outcome sets.^13^ Second, previous models extracted only a small number of variables,^9^^–^ ^11^ and whether the reported performance can be extended to the full set of data commonly extracted in an SR is unclear. Third, methods reported so far in the literature often rely on iterative human-to-computer interactions with the LLM models, a process that can be highly time-consuming for large-scale reviews. For instance, extracting 50 variables from 20 randomized controlled trials (RCTs) would require up to 1,000 manual interactions.^10^^,^ ^11^ Assuming 30 seconds per interaction, it takes over 80 hours. Finally, the lack of open-source code and data for many of these systems hinders widespread adoption and improvement.^13^

The primary objective of this study was to develop and evaluate an open-source system that can automatically perform DE tasks within the context of SRs. “Automatically” refers to the absence of manual interaction from data input to output. We used the protocol and DE manual of a published SR and component network meta-analysis (NMA) on cognitive behavioral therapy for insomnia^14^ to generate a set of meta-prompts via GPT-4o, including explanations for each variable. We then evaluated the performance of several DE methods using these prompts in the dataset for this NMA by GPT-4o. Finally, we assessed the external validity of our system using an updated search dataset of the NMA and another dataset from a published automated DE study.^10^

## 2 Methods

### 2.1 Study process

**Figure 1:**
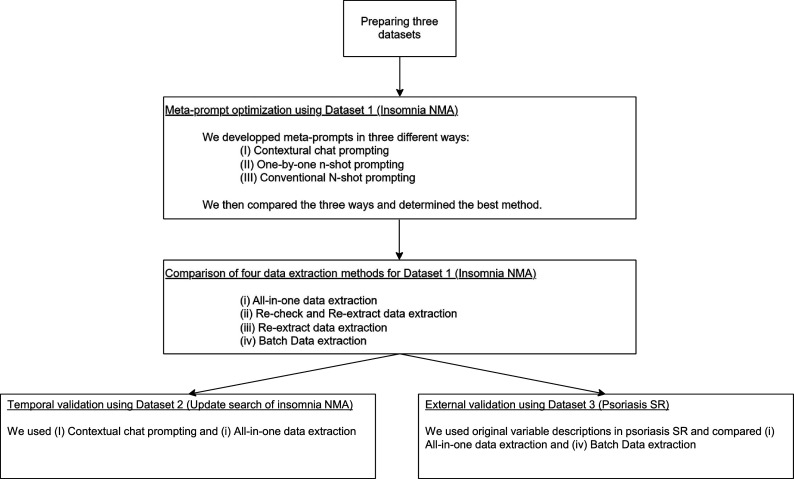
Study process overview. The flowchart depicted in this figure illustrates the overall process of development and external validation of our automated data extraction system. Meta-prompt: a set of instructions given to the large language model (LLM) to instruct it to perform a specific task. Prompting: the process of providing a meta-prompt and input to an LLM to retrieve a desired output. For detailed explanations of individual methods and techniques, please refer to the corresponding sections in Section 2.

Figure 1 illustrates the entire study process. A prospectively registered protocol was not prepared because the study followed an iterative ML development cycle that required ongoing system refinement.

We used a meta-prompt strategy to enhance the LLM.^15^ A prompt is input text given to the LLMs by users. For instance, “What is the weather like today?” In contrast, a meta-prompt is a prompt that tells the LLMs how to perform a specific task. For instance, a meta-prompt might be: “Extract the sample size from this article. Ensure that you extract the total number of participants recruited at the start, rather than the number in each arm, or the number that appeared in the analysis:{article}.”

**Figure 2:**
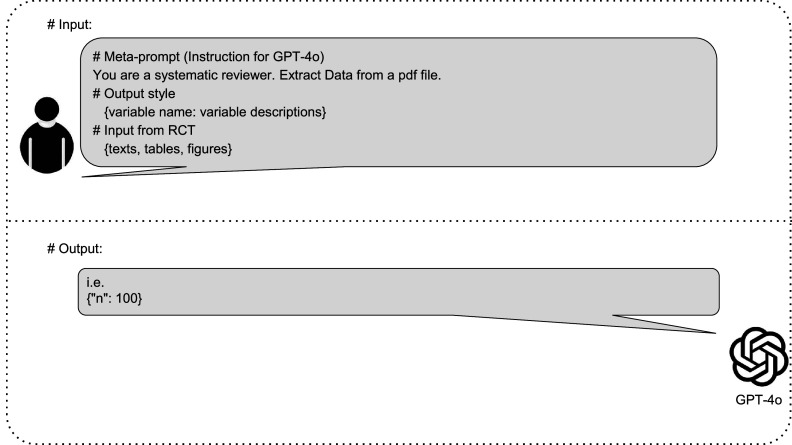
Schematic representation of GPT-4o-based data extraction (DE) process for systematic reviews. This figure illustrates the input provided to GPT-4o and the corresponding response in the context of RCT DE. The input section shows a meta-prompt containing instructions for GPT-4o, along with specifications for the output style, including variable names accompanied by their descriptions, as well as sample RCT data encompassing text, tables, and figures. The response section demonstrates the structured output format that GPT-4o uses to present the extracted data.

The meta-prompt approach can improve model performance without requiring changes to the LLM itself. First, the implementation of meta-prompts is more cost-effective than alternative ways of improving LLM performance, which may require re-training (or “fine-tuning”) the model itself. Second, meta-prompts can be easily adapted to new, superior LLMs as they emerge. Figure 2 illustrates the schema of the DE task.

**Figure 3:**
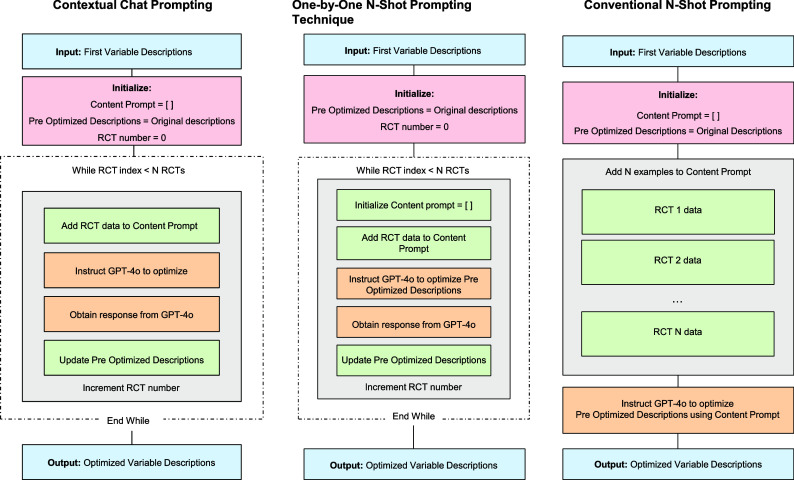
Three prompting techniques to optimize meta-prompts. This figure illustrates three different methods for optimizing meta-prompts, including variable descriptions using GPT-4o. Each method starts with the first variable descriptions as input and processes RCT data differently to generate optimized meta-prompts. The contextual chat and one-by-one methods iterate through RCTs individually, whereas the conventional method processes all RCT data at once.

We first prepared the three datasets for the study (Section 2.2). Then, we used GPT-4o to create and optimize DE meta-prompts using Dataset 1. To optimize our meta-prompts, including the variable descriptions to be used in the automated DE, we compared three prompting techniques (Figure 3). We applied a 10-fold cross-validation to obtain realistic performance measures (Section 2.3). Once the best-performing meta-prompts were selected, we further improved them by correcting the apparent discrepancies between the GPT-4o-obtained data and the human-extracted data. Then, we compared four DE methods using these improved meta-prompts (Section 2.4).

Lastly, we evaluated the external validity of our system on Datasets 2 and 3 (Section 2.5).

### 2.2 Dataset preparation

We used three datasets for this study. Dataset 1 included all 290 RCTs included in the NMA.^14^ Dataset 2 included five RCTs from the updated search for the NMA from which Dataset 1 was drawn. Dataset 3 included 10 RCTs from an SR of targeted immune modulators of psoriasis, which have also been used in a previous study to automate DE.^10^

In addition to the RCTs, Dataset 1 included the study protocol for the NMA, a DE manual, and a spreadsheet containing all the extracted data from the primary publications of the RCTs. In addition, Dataset 1 included PDF files and web links for the trial registry of each RCT. We downloaded the content of the study registry for each trial. Multiple publications related to the same trial were retrieved and linked.

Dataset 2 comprises five studies identified as a result of an updated search for the review in Dataset 1. Dataset 2 utilized the same study protocol, DE manual, and DE methodology as Dataset 1. We prepared Dataset 2 by replicating the original search on PubMed on June 17, 2024. We found 240 abstracts indexed since the date of the previous search. After the title and abstract screening was completed by two independent reviewers, 73 abstracts moved to the full-text screening phase. We randomly sampled articles with available results from candidate full-text articles. Two independent reviewers assessed each sampled article sequentially. We continued this process until we identified five eligible RCTs. We selected five RCTs for validation, matching the number used in Dataset 1. Two reviewers conducted DE independently, using the same DE schema as the original review. Any disagreements were resolved through discussion.

Dataset 3 consisted of the PDFs of all 10 RCTs and the corresponding answers included in the previous semi-automatic DE study.^10^^,^ ^11^ We used exactly the same dataset as in this previous study of automated DE to ensure comparability.

We used the Adobe PDF Extract application programming interface (API) ^16^ to divide the PDF files into main text, tables, and figures. Due to the large number of pages, the Adobe API could not extract the appropriate text for eight RCTs in Dataset 1. In the ensuing analyses, we used the information for these RCTs, excluding the text, because the data sources, such as the corresponding human-extracted data, were already available for them.

### 2.3 Development of meta-prompts

To develop meta-prompts that extract data for the NMA, we input both an initial meta-prompt (the instruction) and the RCT data to be processed into GPT-4o. These data included main texts, tables converted to Excel files, and figures converted to a common raster image extension (i.e., PNG) from the RCT articles in Dataset 1. Additionally, we input results extracted by GPT-4o from the RCT articles using the pre-developed meta-prompt and the corresponding human-extracted data as the reference standard. For coding consistency, we developed meta-prompts using all available information, even if the manuscript data were not processed as text files or human references were missing.

#### 2.3.1 Choosing and improving the meta-prompt

We used a 10-fold cross-validation approach to internally validate our results. For each fold of the cross-validation, we randomly divided 290 RCTs in Dataset 1 into 261 RCTs for development and 29 RCTs for evaluation. Due to the input word count limitations of GPT-4o, we were able to input only up to 5 RCTs out of 261 RCTs for development or out of 29 RCTs for evaluation. We varied the number of RCTs in the development dataset from 0 to 5 in the hope of finding the optimal number and thereby reducing the costs of using GPT-4o. We used 5 RCTs randomly selected out of the 29 RCTs for evaluation without replacement. Once randomly selected out of the 261 or the 29, the same RCTs were used consistently across all three prompting techniques within each fold. The details of three prompting techniques are explained in Section 2.3.2.

In our study, we determine “required variables” based on human assessment. We defined the following variables for performance measures:

True Positive (TP): When the GPT-4o correctly identifies a required variable AND extracts the correct value.

False Positive Type 1 (FP_1_): When the GPT-4o identifies a required variable but extracts an incorrect value.

False Positive Type 2 (FP_2_): When the GPT-4o extracts a variable that is not required.

False Negative (FN): When the GPT-4o fails to extract a required variable.

True Negative (TN): When the GPT-4o correctly identifies a variable as not required.

Using these variables, we calculated the following metrics:

Sensitivity = TP/(TP + FP_1_ + FN).

Specificity = TN/(TN + FP_2_).

Variable detection comprehensiveness = (TP + FP1)/(TP + FP1 + FN).

Precision = TP/(TP + FP_1_ + FP2).

Accuracy = (TP + TN)/(TP + FP_1_ + TN + FP_2_ + FN).

**Figure 4:**
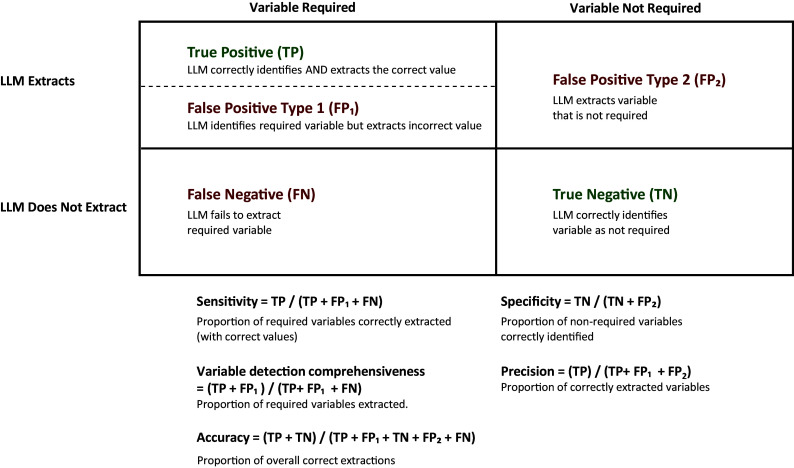
Data extraction evaluation metrics. This figure illustrates the metrics used to evaluate the system. LLM, large language model.

Variable detection comprehensiveness measures the model’s attempt to extract required variables regardless of correctness. Figure 4 illustrates the relationship between these metrics in the context of our DE evaluation framework.

For numerical variables, we considered exact matches as accurate. For string variables, two independent human reviewers visually assessed the semantic equivalence of extracted data and reference standards. Any disagreements were resolved through discussion. Throughout this process and the subsequent process, we calculated mean metrics separately for numeric variables, string variables, and all variables combined.

During the internal validation process, we excluded from the denominator any RCTs where appropriate article data could not be extracted from the PDF. If all RCTs were excluded, we did not use the fold. To prioritize accuracy, we selected the prompting techniques with the best performance for the next stage of modification. Once the optimal variable descriptions were identified, we checked the causes of the extraction errors and modified the descriptions where necessary. Additionally, we adjusted the criteria for correctness to align with human intent within SRs.

#### 2.3.2 Three prompting techniques to optimize meta-prompts

**Figure 5:**
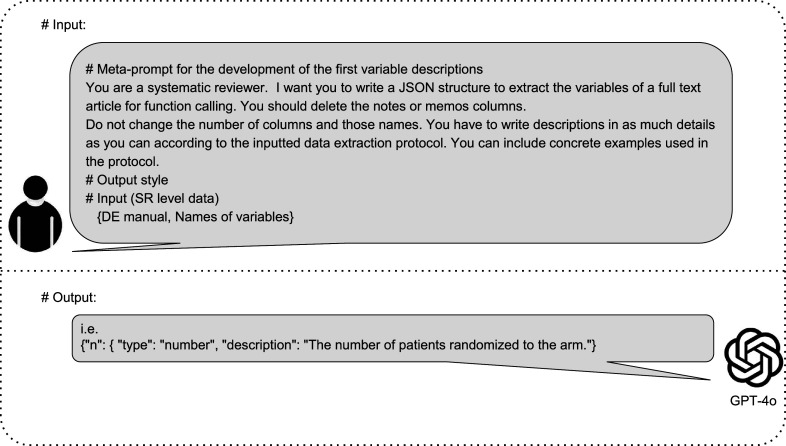
Development of the first meta-prompt (variable description). This figure outlines the process for creating the first variable descriptions for data extraction (DE) in systematic reviews (SRs). The inputs provided to GPT-4o included a meta-prompt with specific instructions for an SR, SR-level data, and a DE manual. The output is an array of objects in a JavaScript object notation (JSON) structure containing variables and their detailed descriptions, generated entirely by GPT-4o based on these inputs. JSON is a simple, structured data format commonly used for text analysis. We adopt a JSON format due to its high representation capacity.

In the first step, we developed meta-prompts that included variable descriptions using the NMA study protocol, the DE manual, and the names of variables from the DE sheet without extracted data (Figure 5). These variables represented the human-extracted information in the NMA, such as study characteristics, population characteristics, and outcomes. We did not include the risk of bias due to the task complexity.^17^ We used three prompting techniques to optimize the meta-prompts (Figure 3). The details are shown in Supplementary Figure 1.

(i) Contextual Chat Prompting: We created a conversational context using one randomly selected paper from the five-RCT subset in the Dataset 1 due to the input constraints. We input GPT-4o prompts structured as a chat, incorporating both the content from the selected paper and relevant meta-prompts. This approach aimed to leverage GPT-4o’s ability to understand the inputs and respond within a dialogue-like framework (Supplementary Table 1).

(ii) One-by-One *N*-Shot Prompting: This stepwise technique began with texts derived from one randomly selected RCT from the five-RCT subset, along with GPT-4o-extracted and human-extracted data. We then applied optimized meta-prompts generated from this initial step to process another randomly selected RCT from the same subset. This iterative process allowed for the gradual refinement of responses (Supplementary Table 1).

(iii) Conventional *N*-Shot Prompting: This comprehensive approach combined messages from multiple RCTs (up to five) from the subset in a single prompt. We supplemented this with GPT-extracted and human-extracted data from all the included RCTs, as well as relevant meta-prompts. This technique aimed to provide GPT-4o with a broader context and more diverse examples in a single interaction (Supplementary Table 1).

### 2.4 Comparison of data extraction methods

We chose one prompting technique based on the above results. In the 10-fold cross-validation, GPT-4o extracted all variables simultaneously (“all-in-one DE”). We explored three other methods based on the concept of re-read prompting.^18^

- Batch DE (to reduce AI hallucinations, i.e., nonsensical or factually incorrect outputs):
  1. Divide the variables into groups of four to reduce the number of variables in a single DE.
  2-1. For each group of four variables, perform batch DE.
  2-2. For variables where GPT-4o determined there were no data in Step 2-1, perform DE for each variable again.
  3. Repeat Step 2 for all groups.
- Re-check and re-extract DE (to improve the sensitivity):
  1. Input all variable descriptions to extract data for all variables.
  2. For variables deemed to have no data, perform a batch check with GPT-4o to determine if data truly do not exist.
  3. For variables where data were extracted in Step 1 and variables found to exist in Step 2, divide the variables into groups of four and perform DE for each group of four.
- Re-extract extracted extraction (to improve the specificity):
  1. Input all variables to extract data for all variables.
  2. For variables where data were extracted in Step 1, divide the variables into groups of four and perform DE for each group of four.

### 2.5 Choosing the best method

From the results of three prompting techniques and four DE methods, we selected the best method. We primarily evaluated based on the accuracy of numeric variables. This is because the accuracy of numeric variables can be assessed objectively without the need for human visual inspection.

### 2.6 External validation in Datasets 2 and 3

#### 2.6.1 Dataset 2

We used the meta-prompts previously developed in Dataset 1 with the modified contextual chat prompting method in the five RCTs (hereafter referred to as the “chat-5-RCT”). For the five RCTs in Dataset 2, we performed DE using 10 different meta-prompts from each fold in the development process. We used the “all-in-one” DE method. We calculated the average accuracy, sensitivity, and specificity using the extracted data in each fold. By evaluating across multiple folds, we intended to capture the variability in performance and provide a more robust estimate of how our method might perform when applied to new, unseen data.

Additionally, we conducted supplementary experiments using the o3 model (released in April 2025) with the same prompts and methodology to assess potential performance improvements.

#### 2.6.2 Dataset 3

For Dataset 3, we used the original authors’ variable descriptions with our meta-prompts. We used the reference standard trial-level results reported by the original authors. Our system extracted data at the arm level, and two independent reviewers evaluated the combined results. We compared the “all-in-one DE” method with the “batch DE” method. The batch method was used to address oversights identified in the “all-in-one approach.”

### 2.7 Development environment

We used Google Collaboratory and the Microsoft Azure OpenAI API (GPT-4o-2024-05-13), as well as the OpenAI API (o3-2025-04-16). The knowledge cutoffs of GPT-4o and o3-2025-04-16 are October 2023 and June 2024, respectively.^19^^,^ ^20^ Dataset 1 is derived from a paper published in January 2024, and this temporal sequence eliminates concerns about potential training data contamination for GPT-4o. The source code is available on GitHub (https://github.com/Tomo-for-lab/automating-DE). We used R Studio (2023.12.1.402.1) with the ggplot2 package (3.5.1) for visualization.^21^

## 3 Results

### 3.1 Development of meta-prompts in Dataset 1

**Figure 6:**
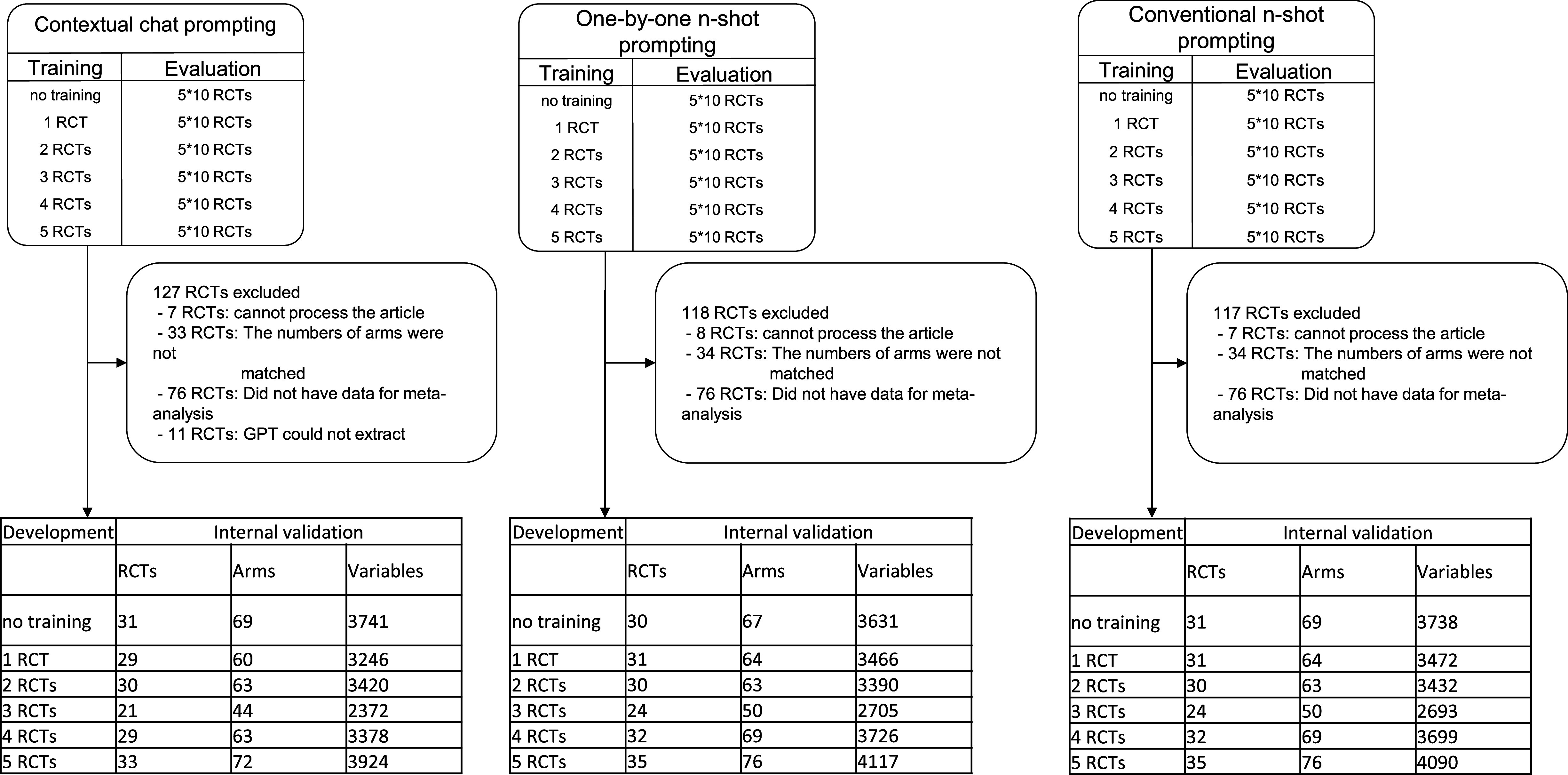
Flowchart of the number of RCTs, arms, and variables examined across different training methods. This figure details the breakdown of RCTs, arms, and variables used in the evaluation of three prompting techniques in 10-fold cross-validation. The top section shows the initial sampling of RCTs for training and evaluation. The middle section details the reasons for excluding various RCTs from the initial sample. API errors occurred when processing articles with many pages, leading to incomplete text extraction. GPT-4o sometimes misidentified the number of trial arms, creating data mismatches. Some trials included in the overall dataset did not undergo data extraction for meta-analysis. The bottom tables present the final counts of RCTs, arms, and variables used in the analysis for each training scenario across the three methods.

Figure 6 presents the detailed number of the sampled RCTs, the included RCTs, the arms in the included RCTs, and the variables examined in Dataset 1.

### 3.2 Performance of three prompting techniques for numeric variable descriptions

We evaluated the performance of the LLM in extracting numeric variables across the three different prompting techniques using 10-fold cross-validation. Table 1 summarizes the results, highlighting sensitivity, specificity, variable detection comprehensiveness, and accuracy for each method and varying numbers of RCTs used for training.

**Table 1:** Performance of three prompting techniques to optimize numeric variable descriptions

		The number of RCTs in the training data					
	Mean (SD)	No training	1 RCT	2 RCTs	3 RCTs	4 RCTs	5 RCTs
Contextual chat prompting	Sensitivity	67.5	69.4	66.3	69.0	67.0	72.7
		(7.46)	(8.22)	(4.79)	(12.9)	(6.65)	(4.93)
	Specificity	64.6	59.1	77.5	64.7	65.5	75.3
		(21.2)	(24.4)	(13.0)	(15.5)	(25.5)	(10.9)
	Precision	63.5	62.6	65.8	63.4	64.3	70.6
		(5.67)	(7.37)	(4.33)	(7.08)	(6.72)	(5.18)
	Variable detection comprehensiveness	96.4	98.1	93.3	96.7	95.5	94.6
		(3.93)	(2.47)	(4.00)	(4.18)	(5.63)	(3.33)
	Accuracy	67.5	67.2	69.6	67.6	67.2	73.4
		(6.12)	(8.73)	(4.46)	(7.78)	(7.34)	(3.75)
One-by-one *n*-shot prompting	Sensitivity	67.4	70.2	66.3	67.8	68.4	72.4
		(7.17)	(7.07)	(6.39)	(14.3)	(6.22)	(6.13)
	Specificity	57.2	59.4	75.1	71.3	62.8	67.1
		(25.8)	(25.0)	(11.0)	(21.2)	(33.4)	(14.8)
	Precision	63.1	64.7	63.3	65.0	67.0	66.9
		(5.89)	(7.25)	(6.38)	(10.3)	(7.38)	(8.13)
	Variable detection comprehensiveness	95.6	96.9	96.7	96.1	94.6	96.1
		(3.65)	(3.88)	(2.19)	(4.13)	(5.76)	(3.73)
	Accuracy	66.3	68.5	68.8	69.1	68.8	70.8
		(6.28)	(8.36)	(5.79)	(11.4)	(7.99)	(6.37)
Conventional *n*-shot prompting	Sensitivity	68.7	69.2	65.9	70.2	67.6	73.2
		(7.08)	(7.02)	(8.15)	(12.0)	(5.25)	(5.65)
	Specificity	62.4	58.8	78.1	67.0	58.9	66.9
		(19.9)	(24.6)	(14.6)	(22.1)	(20.8)	(18.6)
	Precision	63.9	62.8	65.9	67.4	63.3	67.6
		(6.17)	(6.83)	(6.35)	(11.1)	(4.06)	(8.38)
	Variable detection comprehensiveness	96.7	97.9	93.3	95.9	96.4	96.8
		(3.55)	(2.56)	(3.42)	(5.30)	(4.09)	(3.81)
	Accuracy	67.8	67.2	70.0	70.0	66.5	71.7
		(6.43)	(8.22)	(5.45)	(11.1)	(4.64)	(6.44)

*Note*: Values are represented as mean (standard deviation). This table presents the sensitivity, specificity, and accuracy of numeric variable extraction across three different methods: contextual chat prompting, one-by-one *n*-shot prompting, and conventional *n*-shot prompting. For each method, the results are presented for varying numbers of training data (0–5 RCTs). The underbar shows the highest accuracy.

Sensitivity ranged from 65.9% to 73.2%, specificity from 57.2% to 78.1%, precision from 62.6% to 70.6%, variable detection comprehensiveness from 93.3% to 98.1%, and accuracy from 66.3% to 73.4%. Regarding accuracy, the contextual chat prompting method achieved the highest accuracy of 73.4% when trained with five RCTs (chat-5-RCT).

### 3.3 Performance of the modified chat-5-RCT method for string variables and all the variables in Dataset 1

Based on the previous experiment, we selected the contextual chat prompting technique with five RCTs due to the highest accuracy (chat-5-RCT). In some cases, variable descriptions in the meta-prompt were changed as the extracted data due to an error by GPT-4o. Hence, when optimizing descriptions, we used GPT-4o to judge whether the output was an optimized meta-prompt or extracted data for each variable. If the output was extracted data, we used a pre-optimized description. When evaluating performance, we ensured alignment with the human-unique extracted data. For example, when extracting data for multiple outcome evaluations, data extractors used a shorthand code of “*” if a scale had already been recorded for a previous outcome, eliminating the need to re-enter the full-scale name.

**Figure 7:**
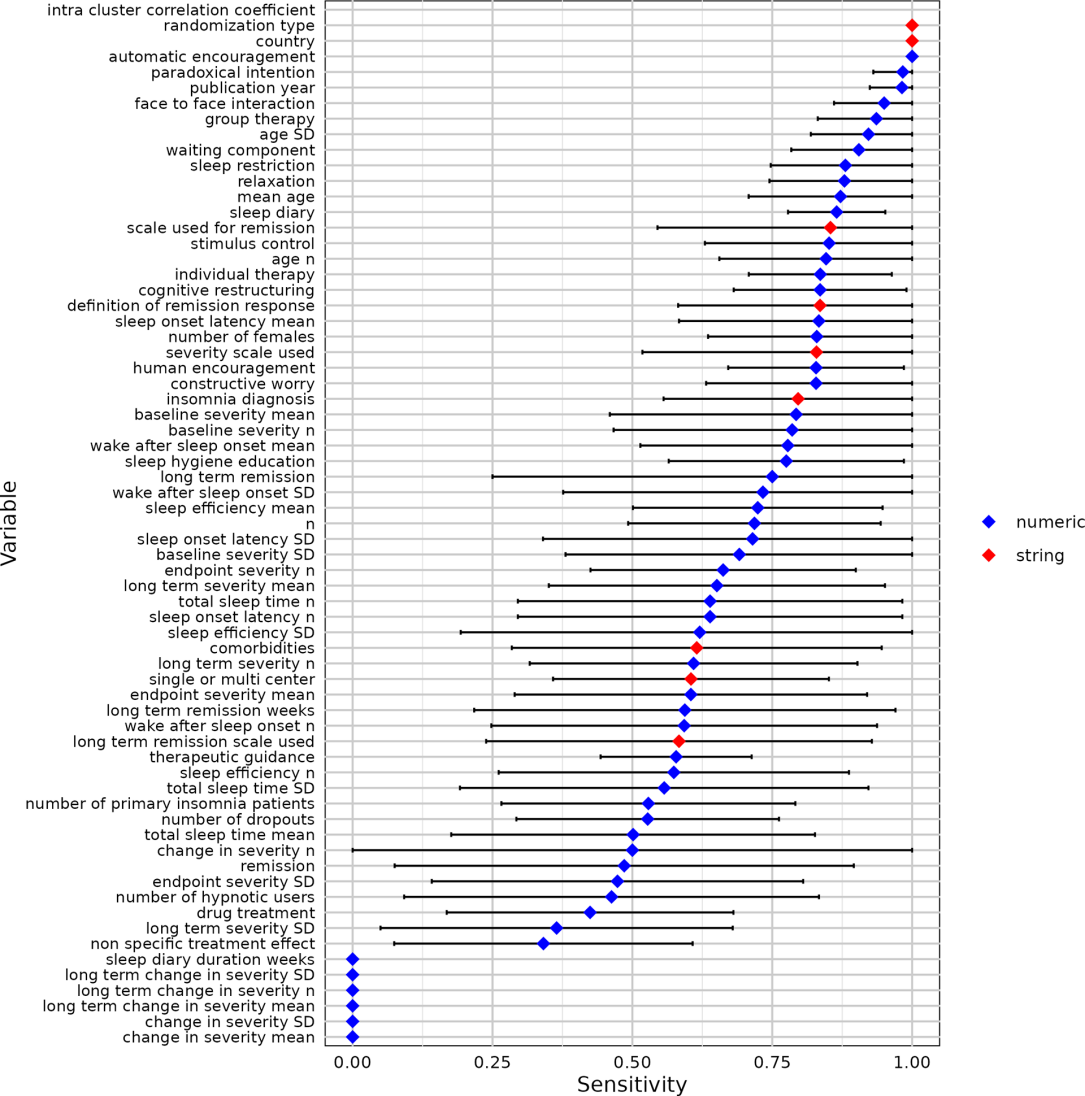
Sensitivity for all variables by the chat-5-RCT method with modifications in Dataset 1. Square: mean. Horizontal line: standard deviation. Some variables lack data points because a human reviewer extracted all relevant information, leaving no examples of “missing” data to calculate specificity against.

**Figure 8:**
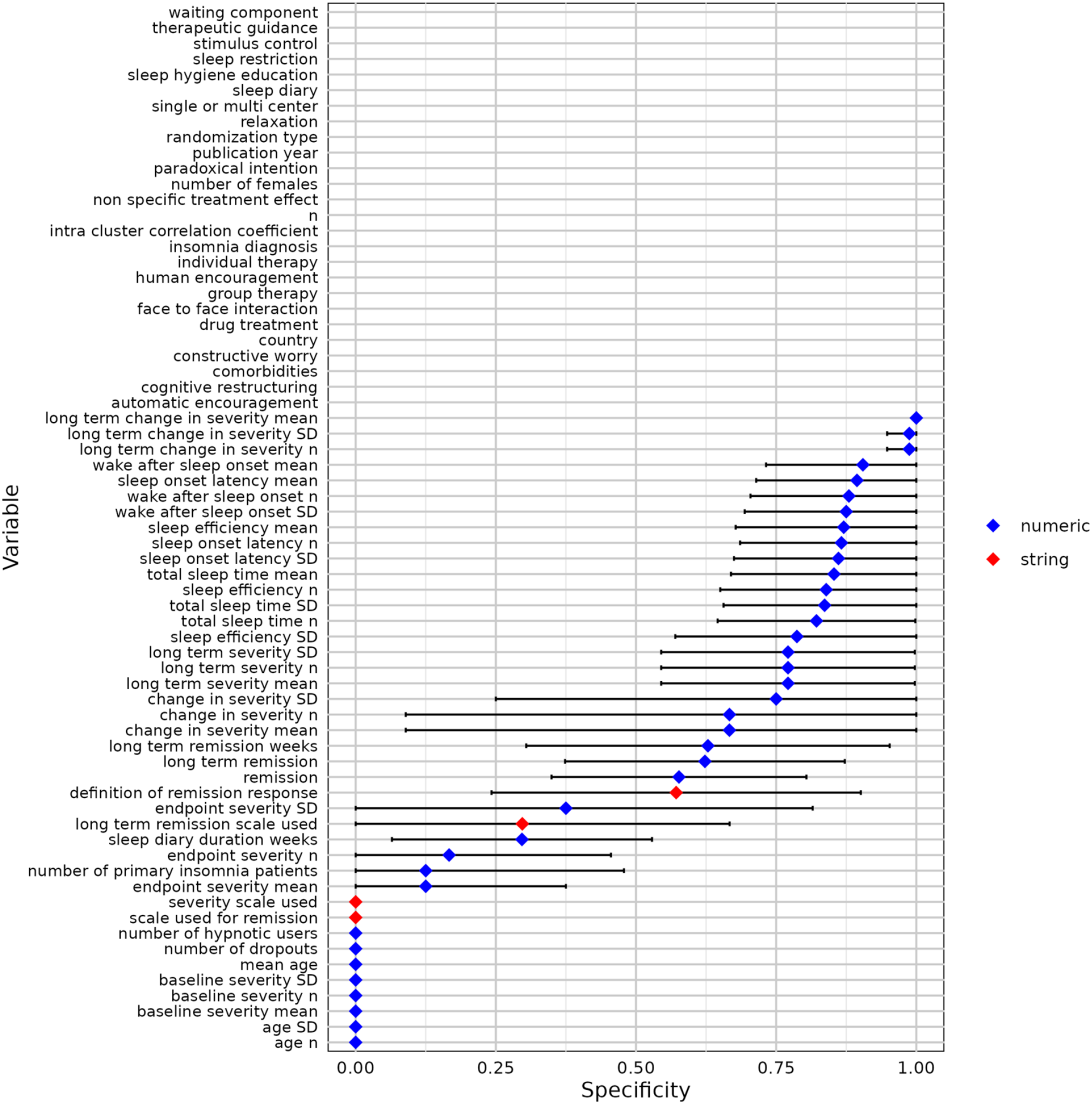
Specificity for all variables by the chat-5-RCT method with modifications in Dataset 1. Square: mean. Horizontal line: standard deviation. Some variables lack data points because a human reviewer extracted all relevant information, leaving no examples of “missing” data to calculate specificity against.

**Figure 9:**
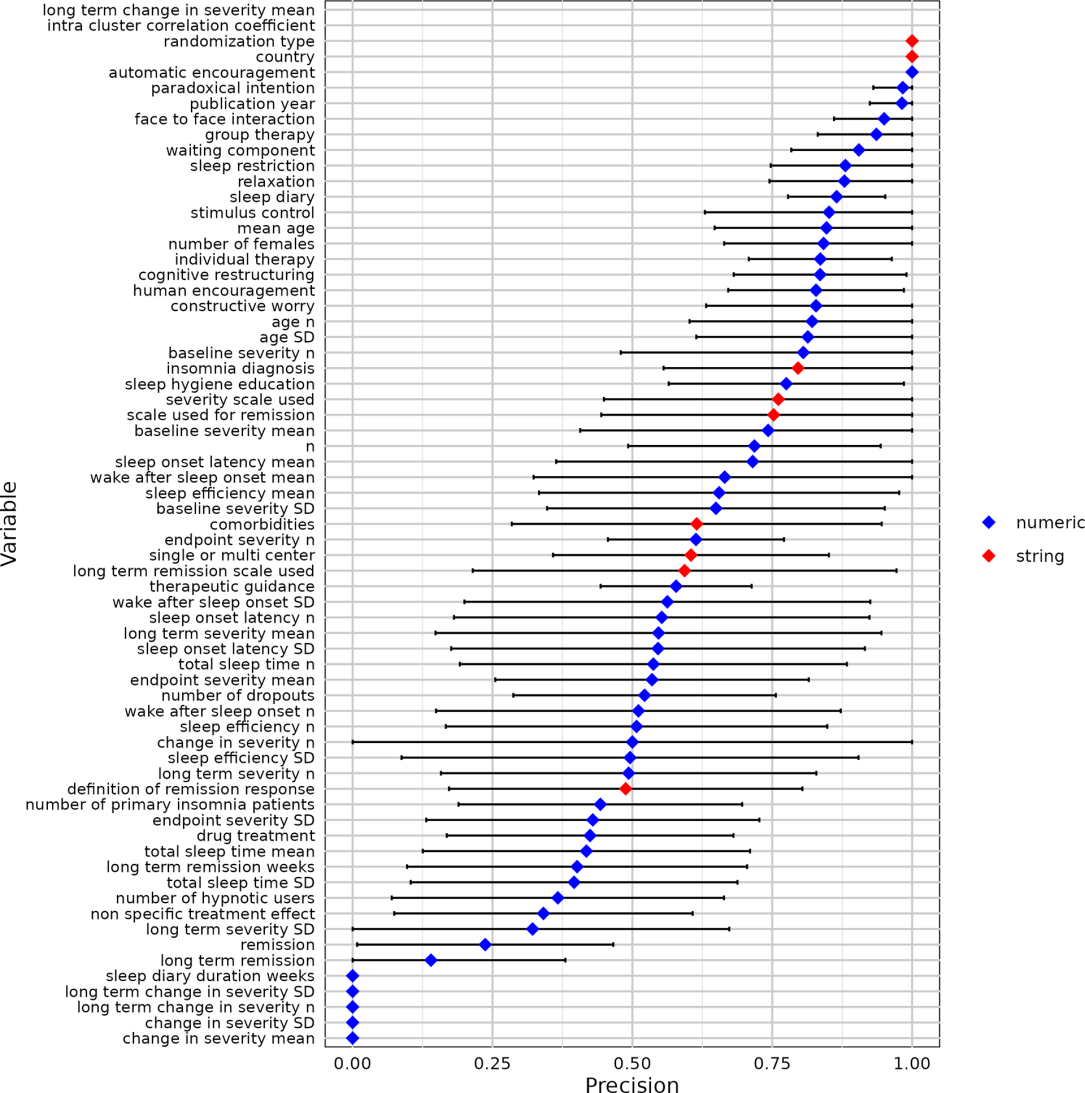
Precision for all variables by the chat-5-RCT method with modifications in Dataset 1. Square: mean. Horizontal line: standard deviation. Some variables lack data points because a human reviewer extracted all relevant information, leaving no examples of “missing” data to calculate specificity against.

**Figure 10:**
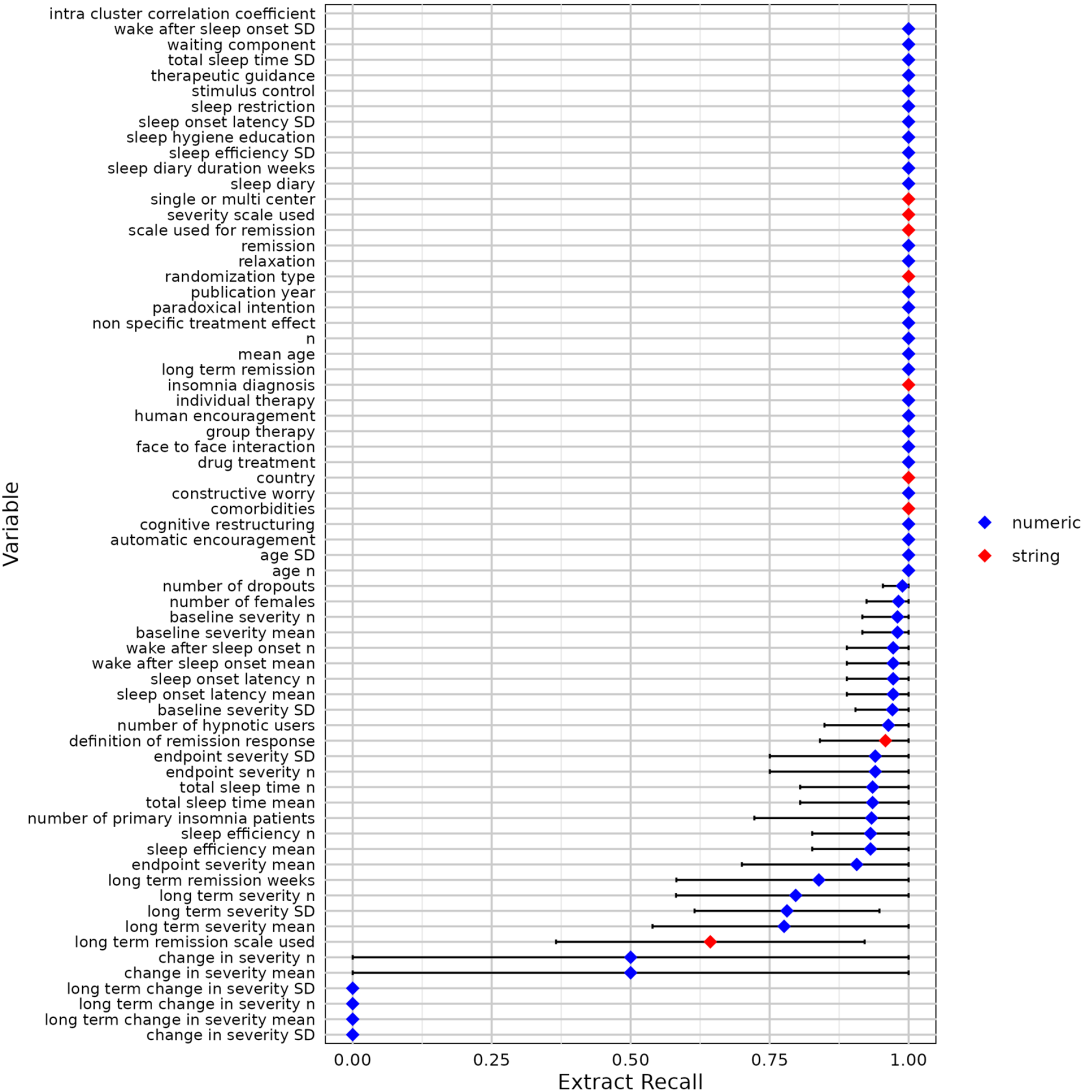
Variable detection comprehensiveness for all variables by the chat-5-RCT method with modifications in Dataset 1. Square: mean. Horizontal line: standard deviation. Some variables lack data points because a human reviewer extracted all relevant information, leaving no examples of “missing” data to calculate specificity against.

**Figure 11:**
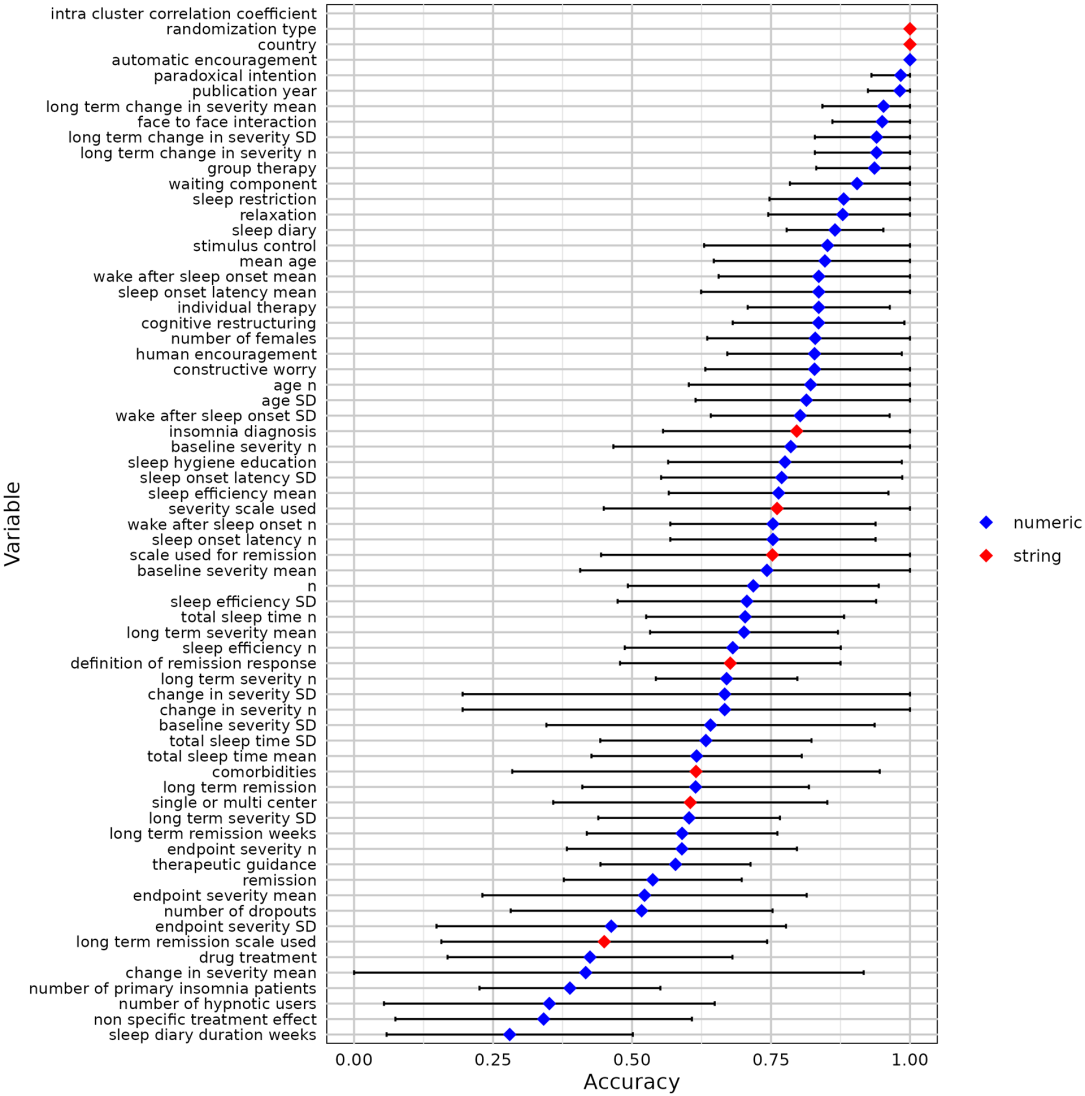
Accuracy for all variables by the chat-5-RCT method with modifications in Dataset 1. Square: mean. Horizontal line: standard deviation.

We used 10-fold cross-validation to evaluate the modified contextual chat prompting method with five RCTs (Table 2). Figures 7–11 show the sensitivity, specificity, precision, variable detection comprehensiveness, and accuracy results for each variable. In the subsequent stage, we evaluated the performance in the modified ways.

**Table 2:** Performance of the chat-5-RCT method with modifications in Dataset 1

	Numeric (58 variables)	Strings (9 variables)	Total (67 variables)
Sensitivity	73.5	79.0	74.4
	(5.32)	(10.8)	(4.87)
Specificity	70.4	50.6	68.8
	(12.1)	(34.8)	(12.6)
Precision	68.0	75.1	69.1
	(8.52)	(8.94)	(7.48)
Variable detection comprehensiveness	97.2	96.6	97.1
	(2.38)	(2.94)	(2.27)
Accuracy	72.3	74.0	72.6
	(6.91)	(7.92)	(6.05)

*Note*: Values are represented as mean (standard deviation). This table presents the sensitivity, specificity, variable detection comprehensiveness, and accuracy for numeric and string variables using the modified contextual chat prompting method with five RCTs.

### 3.4 Comparison of data extraction methods in Dataset 1

Using the “chat-5-RCT” prompting technique, we compared four DE methods in Dataset 1 (Table 3). The all-in-one DE method achieved the highest accuracy of 72.3% (SD 6.91) with sensitivity of 73.5% (SD 5.32), specificity of 70.4% (SD 12.1), precision of 68.0% (SD 8.52), and variable detection comprehensiveness of 97.2% (SD 2.38). The batch DE method had the lowest accuracy of 54.9% (SD 7.79), with sensitivity of 72.8% (SD 5.13) and low specificity of 2.76% (SD 2.99). The re-extract method showed comparable accuracy at 71.9% (SD 7.61), with sensitivity of 70.8% (SD 5.89), the highest specificity of 76.1% (SD 17.3), precision of 68.1% (SD 9.04), and variable detection comprehensiveness of 95.5% (SD 4.90). The re-check and re-extract method reached 68.2% (SD 6.72) accuracy, with sensitivity of 71.4% (SD 5.74), specificity of 60.3% (SD 12.9), precision of 63.4% (SD 7.69), and variable detection comprehensiveness of 98.4% (SD 1.21).

**Table 3:** Comparison of prompting techniques for data extraction in Dataset 1

	All-in-one			
	data	Batch	Re-check and	Re-extract
	extraction	data	re-extract data	data
Metric	(original)	extraction	extraction	extraction
Sensitivity	73.5	72.8	71.4	70.8
	(5.32)	(5.13)	(5.74)	(5.89)
Specificity	70.4	2.76	60.3	76.1
	(12.1)	(2.99)	(12.9)	(17.3)
Precision	68.0	54.6	63.3	68.1
	(8.52)	(7.89)	(7.69)	(9.04)
Variable detection comprehensiveness	97.2	100	98.4	95.5
	(2.38)	(0.00)	(1.21)	(4.90)
Accuracy	72.3	54.9	68.2	71.9
	(6.91)	(7.79)	(6.72)	(7.61)

*Note*: Values are represented as mean (standard deviation).

### 3.5 Evaluation in Dataset 2

Table 4 shows the all-in-one DE method for all variables in Dataset 2 using 10-fold cross-validation. Across all variables, GPT-4o showed 61.6% accuracy (SD 1.76), 61.9% sensitivity (SD 2.44), 60.1% specificity (SD 8.99), 61.2% precision (SD 2.86), and 92.2% variable detection comprehensiveness (SD 3.62). For numeric variables specifically, GPT-4o showed 60.4% accuracy (SD 1.85), 60.6% sensitivity (SD 2.33), 59.5% specificity (SD 7.78), 59.5% precision (SD 3.07), and 91.9% variable detection comprehensiveness (SD 3.89). For string variables, GPT-4o showed higher performance with 68.8% accuracy (SD 2.06), 69.0% sensitivity (SD 4.16), 67.0% specificity (SD 31.6), 70.5% precision (SD 2.44), and 93.9% variable detection comprehensiveness (SD 3.25).

**Table 4:** All-in-one data extraction method in Dataset 2

	GPT-4o			o3		
	Numeric	Strings	All	Numeric	Strings	All
Metric	variables	variables	variables	variables	variables	variables
Sensitivity	60.6	69.0	61.9	73.0	84.8	74.9
	(2.33)	(4.16)	(2.44)	(1.54)	(3.94)	(1.07)
Specificity	59.5	67.0	60.1	78.5	57.0	76.7
	(7.78)	(31.6)	(8.99)	(9.13)	(24.1)	(8.05)
Precision	59.5	70.5	61.2	74.2	83.2	75.7
	(3.07)	(2.44)	(2.86)	(2.43)	(2.96)	(2.29)
*Variable detection comprehensiveness*	91.9	93.9	92.2	92.9	96.8	93.5
	(3.89)	(3.25)	(3.62)	(2.81)	(2.37)	(2.53)
Accuracy	60.4	68.8	61.6	74.2	81.7	75.3
	(1.85)	(2.06)	(1.76)	(1.48)	(2.41)	(1.34)

*Note*: Values are represented as mean (standard deviation).

For o3, the performance improved substantially over GPT-4o. Across all variables, o3 showed 75.3% accuracy (SD 1.34), 74.9% sensitivity (SD 1.07), 76.7% specificity (SD 8.05), 75.7% precision (SD 2.29), and 93.5% variable detection comprehensiveness (SD 2.53). Focusing on numeric variables only, o3 showed 74.2% accuracy (SD 1.48), 73.0% sensitivity (SD 1.54), 78.5% specificity (SD 9.13), 74.2% precision (SD 2.43), and 92.9% variable detection comprehensiveness (SD 2.81). For string variables, o3 showed 81.7% accuracy (SD 2.41), 84.8% sensitivity (SD 3.94), 57.0% specificity (SD 24.1), 83.2% precision (SD 2.96), and 96.8% variable detection comprehensiveness (SD 2.37).

### 3.6 Evaluation in Dataset 3

Table 5 presents a comparison of the performance of our “all-in-one DE” method and the “batch DE” method in Dataset 3 with the results obtained in another study using Claude 2.^10^ The “all-in-one DE” method using GPT-4o had a mean accuracy of 84.4%. In contrast, the batch DE method using the modified GPT-4o approach achieved a mean accuracy of 96.3%.

**Table 5:** Comparison of the data extraction method in Dataset 3

	Gartlehner Claude 2 accuracy	All-in-one data extraction GPT-4o accuracy	Batch data extraction GPT-4o accuracy
First author, last name	100% (10/10)	100% (10/10)	100% (10/10)
Trial registry number	90% (9/10)	100% (10/10)	100% (10/10)
Study name, acronym	100% (10/10)	80% (8/10)	90% (9/10)
Study funder	100% (10/10)	100% (10/10)	100% (10/10)
Mean age	90% (9/10)	80% (8/10)	100% (10/10)
Female participants	100% (10/10)	70% (7/10)	90% (9/10)
Mean PASI score at baseline	100% (10/10)	70% (7/10)	100% (10/10)
Mean duration of disease	90% (9/10)	60% (6/10)	90% (9/10)
Inclusion criteria	100% (10/10)	100% (10/10)	100% (10/10)
Exclusion criteria	90% (9/10)	90% (9/10)	100% (10/10)
*N* randomized	100% (10/10)	100% (10/10)	100% (10/10)
*N* randomized per group	100% (10/10)	100% (10/10)	100% (10/10)
*N* analyzed	100% (10/10)	20% (2/10)	90% (9/10)
Dose, route, and frequency of intervention	100% (10/10)	90% (9/10)	100% (10/10)
Primary outcome	100% (10/10)	100% (10/10)	90% (9/10)
Primary outcome, effect estimate	80% (8/10)	90% (9/10)	90% (9/10)

*Source:* Gartlehner et al. (2024).^10^ *Note*: This table compares the accuracy of data extraction (DE) from Dataset 3 using three different methods: Gartlehner (with Claude 2), all-in-one DE (with GPT-4o), and batch DE (with GPT-4o).

## 4 Discussion

We developed and evaluated an automated system for DE in SRs using GPT-4o and o3. Our results demonstrated varying levels of performance across different datasets and methods. In Dataset 1, we found that the contextual chat prompting method with five RCTs (chat-5-RCT) showed the highest accuracy of 73.4% among three optimization methods. After modifications, our evaluation on Dataset 1 achieved 72.3% accuracy, 73.5% sensitivity, 70.4% specificity, 68.0% precision, and 97.2% variable detection comprehensiveness across all variables. Our evaluation on Dataset 2 GPT-4o demonstrated slightly lower performance with 61.6% accuracy, 61.9% sensitivity, 60.1% specificity, 61.2% precision, and 92.2% variable detection comprehensiveness for all variables. For o3, performance improved substantially over GPT-4o. Across all variables, o3 showed 75.3% accuracy, 74.9% sensitivity, 76.7% specificity, 75.7% precision, and 93.5% variable detection comprehensiveness. Notably, in Dataset 3, which has few missing variables, we found that the “batch DE” method achieved a mean accuracy of 96.3%, comparable to the previous study using Claude 2 with manual interaction (96.3%).^10^

Our results suggest the potential utility of our system for replacing one of two independent human reviewers for extracting string variables. Unlike previous studies that required iterative interaction from the end user,^10^^,^ ^11^ our approach omitted the need for human interaction during the extraction process. For string variables, our batch extraction method achieved good accuracy on Dataset 3, comparable to another study using Claude 2 with a manual interaction.^10^ The high variable detection comprehensiveness we observed for string variables in Datasets 1 and 2 further supports this potential. The results were on a par with those from other studies.^13^ These results suggest that systematic reviewers could use our system in SR to reduce the time spent manually checking for missed information when extracting string variables.

In contrast to string variables, our results showed that numeric variable extraction performed poorly. This finding aligns with previous research highlighting the challenges of extracting quantitative data using LLMs.^22^ The risk of generating incorrect numerical values remains a concern. Recent work has explored potential solutions such as retrieval-augmented generation techniques, which aim to improve output accuracy by providing LLMs with processed source documents.^12^^,^ ^23^ We input plain texts and figures into GPT-4o and improved the meta-prompt. Future studies should explore alternative input methods to achieve further improvements.

This study has several limitations. First, we externally evaluated our system on only two datasets, which may limit the applicability of our findings to other SR topics. Further investigation in different fields of study or types of reviews will be necessary. Second, while our system is open-source, it relies exclusively on GPT-4o, a model whose detailed internal structure is not publicly accessible. This black-box nature poses a risk that results might change in the future, as the GPT-4o may be updated without notice. Consequently, continuous accuracy verification using standardized benchmarks is necessary to ensure consistent performance over time. Additionally, the rapid pace of development in LLMs means that newer models may soon outperform the GPT-4o used in this study. Fourth, GPT-4o is a widely used but expensive model, which limits the implementation of our system to those who can afford it. This cost barrier may restrict the broader adoption and replication of our research. It’s worth noting that the core methodology of our approach is not intrinsically tied to GPT-4o and could potentially be adapted to other language models, including open-source alternatives, with minor adjustments. Future work could explore the use of more accessible models to increase the applicability and reproducibility of this research. Fifth, some reference variables contained data unavailable from the full text but originally obtained by direct requests to the study authors. However, this information bias could reduce system performance.

In conclusion, we developed a fully automated system where humans only need to input the SR protocol and variable definitions (users do not need to write the prompts themselves). All the steps covered in this article are open access (https://github.com/Tomo-for-lab/automating-DE), so that other researchers can replicate our findings, apply them to their own SRs and data, and further improve/adapt the methods. Additionally, our system extracted data directly from primary research articles in the context of a real SR using a large-scale dataset, reflecting the authentic challenges and complexities encountered in SRs.

## Supporting information

Kataoka et al. supplementary material Kataoka et al. supplementary material

## Data Availability

The data that support the findings of this study are openly available at https://github.com/Tomo-for-lab/automating-DE.

## Supplementary material

To view supplementary material for this article, please visit http://doi.org/10.1017/rsm.2025.10030.
