# Supplementary material for: Automating the data extraction process for systematic reviews using GPT-4o and o3
Source: Res Synth Methods. 2025 Sep 17;17(1):42–62. doi: 10.1017/rsm.2025.10030 (PMC12823200; doi:10.1017/rsm.2025.10030)

Supplemental table 1. Schematic conversations of three prompting techniques

| Prompting techniques | Speaker | Dialogue |
| --- | --- | --- |
| Contextual Chat Prompting Technique | Researcher | Let's start our Contextual Chat Prompting process. Here's a snippet from an RCT we're analyzing: "A total of 100 patients were enrolled in the study, with 50 randomly assigned to the treatment group and 50 to the control group." The data you extracted: n1 (treatment group) = 50, n2 (control group) =50, Correctly extracted data: n = 50 in each arm.  Based on this example, please provide an optimized description for the variable 'n': the total number of participants. |
|  | GPT-4o | Certainly. Based on the provided information, I can optimize the description for the variable "n" as follows:  {"n": { "type": "number", "description": "The number of patients randomized to each arm of the study. This represents the initial allocation of participants to either the treatment or control group, regardless of subsequent dropouts or protocol deviations. For example, in a study with 100 total participants where 50 were assigned to the treatment group and 50 to the placebo group, 'n' would be 50 for each arm." }} |
|  | Researcher | That's good. Now, let's look at another part of the study: "At the 6-month follow-up, 45 patients in the treatment group and 48 in the placebo group completed the assessment. However, all 100 initially randomized patients were included in the final analysis." How might this information further refine our description of "n"? ."  Based on the previous extractions we've made, please continue to extract the next data point while considering the context we've established. |
|  | GPT-4o | Thank you for providing that additional context. I can further refine the description of "n" to clarify the difference between initial allocation and follow-up numbers:  {"n": { "type": "number", "description": "The number of patients initially randomized to each arm of the study at the beginning of the trial. This represents the original allocation of participants to either the treatment or control group, regardless of subsequent dropouts or protocol deviations. It's important to note that 'n' refers to the initial number, not the number of participants who completed the study. For example, in a study where 50 participants were initially assigned to each group, 'n' would be 50 for each arm, even if only 45 in the treatment group and 48 in the placebo group completed the follow-up assessment." }} |
| One-by-One N-Shot Prompting | Researcher | Let's start our One-by-One N-Shot Prompting process. We'll optimize our variable descriptions using data from multiple RCTs, one at a time.  Let's begin with the first RCT in our dataset. Here's an excerpt: 'The study included 200 participants, with 100 in the intervention group and 100 in the control group.' Correctly extracted data: n = 100 in each arm.  How should we update our description for the variable 'n': the total number of participants.? |
|  | GPT-4o | Based on this information, I suggest updating the description for 'n' as follows:  {'n': { 'type': 'number', 'description': 'The number of participants allocated to each arm of the study at the beginning of the trial. For instance, in a study with 200 total participants equally divided between intervention and control groups, 'n' would be 100 for each arm.' }} |
|  | Researcher | Let's start our One-by-One N-Shot Prompting process. We'll optimize our variable descriptions using data from multiple RCTs, one at a time.  Let's begin with the first RCT in our dataset. Here's an excerpt: ‘This study reports: '150 patients were randomized, and all were included in the intention-to-treat analysis, including 5 who withdrew consent after receiving treatment.' Correctly extracted data: n = 75 in each arm.  How should we update our description for the variable 'n': 'The number of participants allocated to each arm of the study at the beginning of the trial. For instance, in a study with 200 total participants equally divided between intervention and control groups, 'n' would be 100 for each arm.’.? |
|  | GPT-4o | This new information helps us refine the description further:  {'n': { 'type': 'number', 'description': 'The number of participants initially randomized to each arm of the study, regardless of subsequent withdrawals or dropouts. For example, in a study where 150 patients were randomized and 5 withdrew consent before treatment, 'n' would still include these 5 patients in the total count.' }} |
| Conventional N-Shot Prompting | Researcher | We're going to use Conventional N-Shot Prompting to optimize our variable descriptions. I'll provide information from multiple RCTs at once, and then we'll use that collective information to update our descriptions.  RCT 1: 'The study included 200 participants, with 100 in the intervention group and 100 in the control group. All 200 were included in the final intention-to-treat analysis.'  Correctly extracted data: n = 100 in each arm.  RCT 2: '150 patients were randomized and included in the intention-to-treat analysis, including 5 who withdrew consent before receiving any treatment.'  Correctly extracted data: n = 150 in each arm.  RCT 3: 'We performed intention-to-treat analysis on all 180 randomized participants, including the 10 who did not complete the study.'  Correctly extracted data: n = 180 in each arm.  RCT 4: 'Of the 250 participants randomized, 230 completed the 12-week follow-up. Intention-to-treat analysis was performed on all 250 randomized participants.'  Correctly extracted data: n = 250 in each arm.  Based on these examples, please provide an optimized description for the variable 'n': the number of participants. |
|  | GPT-4o | Thank you for providing these examples. Based on the information from all four RCTs, I propose the following optimized description for the variable 'n':  {'n': { 'type': 'number', 'description': 'The number of participants initially randomized to each arm of the study, typically used in intention-to-treat (ITT) analysis. This count remains constant regardless of subsequent withdrawals, dropouts, or the number of participants who complete the study. ' }} |

Schematic conversations of three prompting techniques. These dialogues are simplified representations designed to illustrate the general flow of the process, rather than exact replications of the actual results. For detailed implementation and precise conversational flows, please refer to the associated GitHub repository (https://github.com/Tomo-for-lab/automating-DE).

Supplemental Figure 1. A schematic presentation of the meta-prompt to optimize variable descriptions for data extraction


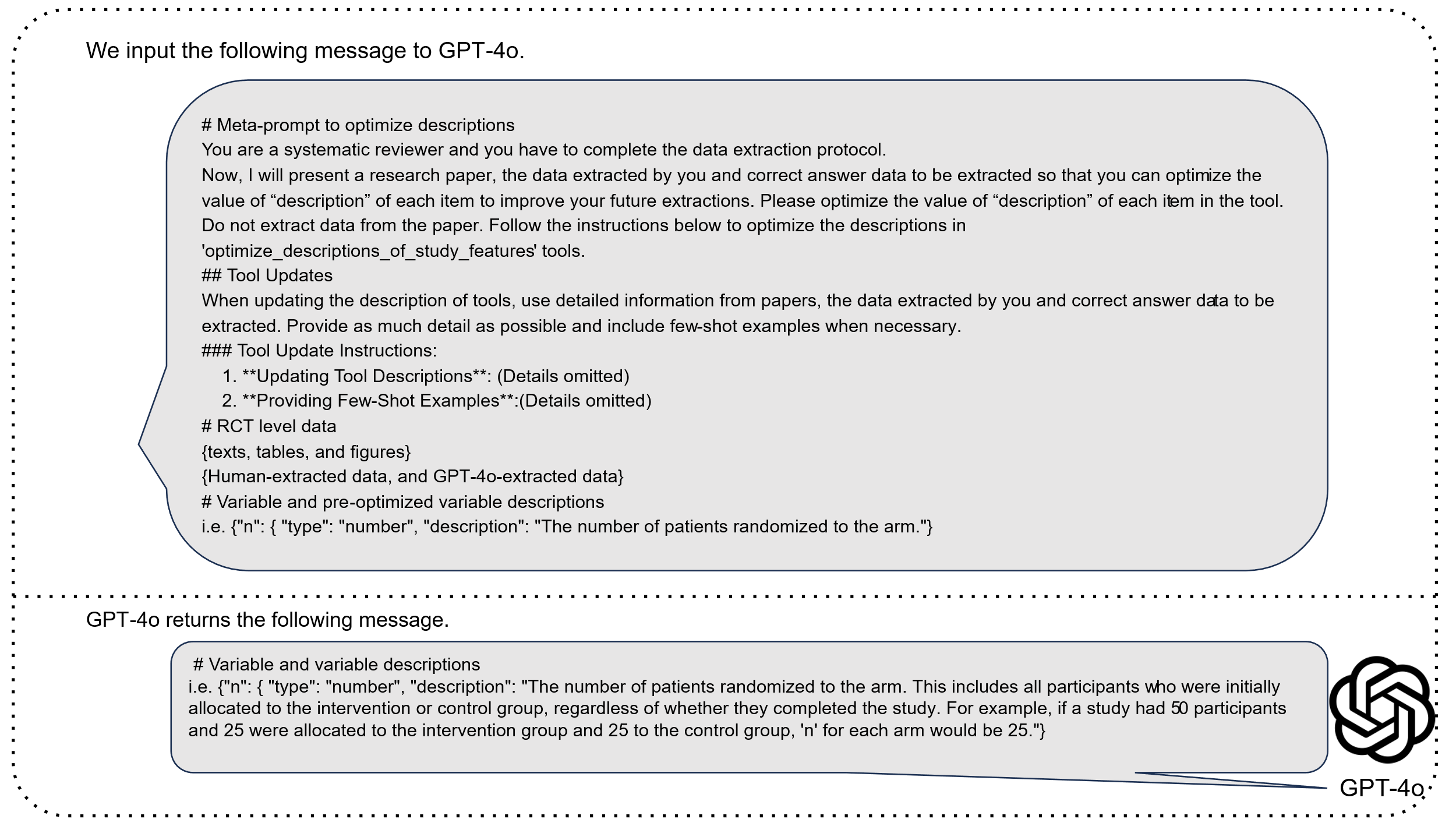

Supplement: Kataoka et al. supplementary material [file S1759287925100306sup001.docx]
